# Cirrhosis of the Liver

**Published:** 1851-09

**Authors:** Jas. S. Hawley

**Affiliations:** Sec’y


ART. V.— *Cirrhosis of the Liver.* Furnished from the Minutes of the Buffalo Medical Association. By JAS. S. HAWLEY, Sec'y. Case reported by Dr. C. H. WILCOX, Aug. 4, 1851.

Was called to see Caroline Ellsworth, in December last. She was twenty years of age, small size; spine curved; sexual system, including the mammæ, entirely undeveloped; had never menstruated; her abdomen somewhat enlarged, though not apparently dropsical. She was suffering from haematemesis at the time, supposed to be vicarious with the menstrual discharge. The blood which she ejected was sometimes fresh, and coagulated after it was thrown out, at other times it was discharged in a coagulated state. This subsided under the use of suitable remedies, in about six hours, after which she was put upon tonics.

January 28, she was re-attacked with hæmatemesis.

February 10, she was attacked with peritonitis, for which she was blistered, and small doses of morphia and calomel administered, by which ptyalism was very readily and unexpectedly produced. About this time she began to show some signs of ascites.

March 1. She had another seizure of vomiting blood, for which mur. tinc. feni was administered, together with small lumps of ice taken into the stomach. This last measure was very grateful to the patient, and very effectual in arresting the hæmorrhage. Digitalis, nitre, and cream of tartar, were given with a view to counteract the dropsical effusion, but it continued to increase, producing great distention and pain, until April 15, when tapping was resorted to, and twenty or twenty-five pounds of fluid evacuated. The abdomen refilled very rapidly, requiring tapping again on the 13th of May. Great irritability of the stomach, accompanied by frequent vomiting of a very acid fluid, came on about the 23d of May, which continued up to the time of her death, on the 8th of June.

Autopsy took place eleven hours after death. Present, Drs. Wilcox, Wallis, Flint, and Hawley. Ascitic distention of the abdomen very great. No anasarca — which circumstance directed attention to the condition of the liver at once. This organ presented to the feel, through the abdomical parietes (after the water had been drawn off) the character of a large nodulated tumor, occupying the right hypochondriac, the epigastric, and part of the left hypochondriac regions. Its whole substance presented a degenerated, compact appearance, paler and less sanguineous, harder and smaller than natural, answering well to that condition known as cirrhosis. It had formed very extensive and firm adhesions to the right kidney, the transverse colon, the stomach, and the diaphragm; besides being attached by thick bands of lymph to the peritoneum. So firm were its adhesions to the adjacent viscera that they necessarily removed together, and afterwards required careful dissections to separate them.

The specimen was then exhibited to the Association, with remarks on the pathology of the case, by Prof. Flint. After alluding to the well-known fact that dropsy depending upon obstruction of the portal system, is confined to the abdomen, he called the attention of the Association to the important circumstance that this patient had in no instance suffered from those diseases which are usually called bilious, and are supposed to depend upon obstruction and torpidity of the liver, although those conditions were eminently present. The discharge of the blood from the stomach was easily explained by the venous engorgement which the obstruction in the liver produced, and so also the serous effusion into the abdomen.

Another interesting feature of the case was the absence of the menstrual function and the corresponding absence of corpora lutea in the ovaries; they being perfectly regular and smooth as they exist before the age of puberty.

